# Comparing public attitudes, knowledge, beliefs and behaviours towards antibiotics and antimicrobial resistance in Australia, United Kingdom, and Sweden (2010-2021): A systematic review, meta-analysis, and comparative policy analysis

**DOI:** 10.1371/journal.pone.0261917

**Published:** 2022-01-14

**Authors:** Olivia Hawkins, Anna Mae Scott, Amy Montgomery, Bevan Nicholas, Judy Mullan, Antoine van Oijen, Chris Degeling

**Affiliations:** 1 Australian Centre for Health Engagement, Evidence and Values, The Faculty of the Arts, Social Sciences and Humanities, University of Wollongong, NSW, Australia; 2 Institute for Evidence-Based Healthcare, Bond University, Queensland, Australia; 3 School of Nursing, Faculty of Science, Medicine and Health, University of Wollongong, NSW, Australia; 4 Illawarra-Shoalhaven Local Health District, NSW Health, Wollongong, NSW, Australia; 5 Centre for Health Research Illawarra Shoalhaven Population, Faculty of Science, Medicine and Health, University of Wollongong, NSW, Australia; 6 School of Medicine, Faculty of Science, Medicine and Health, University of Wollongong, NSW, Australia; 7 Molecular Horizons, Faculty of Science, Medicine and Health, University of Wollongong, NSW, Australia; University of Lincoln, UNITED KINGDOM

## Abstract

**Background:**

Social and behavioural drivers of inappropriate antibiotic use contribute to antimicrobial resistance (AMR). Recent reports indicate the Australian community consumes more than twice the defined daily doses (DDD) of antibiotics per 1000 population than in Sweden, and about 20% more than in the United Kingdom (UK). We compare measures of public knowledge, attitudes and practices (KAP) surrounding AMR in Australia, the UK and Sweden against the policy approaches taken in these settings to address inappropriate antibiotic use.

**Methods:**

National antimicrobial stewardship policies in Australia, Sweden, and the UK were reviewed, supplemented by empirical studies of their effectiveness. We searched PubMed, EMBASE, PsycINFO, Web of Science and CINAHL databases for primary studies of the general public’s KAP around antibiotic use and AMR in each setting (January 1 2011 until July 30 2021). Where feasible, we meta-analysed data on the proportion of participants agreeing with identical or very similar survey questions, using a random effects model.

**Results:**

Policies in Sweden enact tighter control of community antibiotic use; reducing antibiotic use through public awareness raising is not a priority. Policies in the UK and Australia are more reliant on practitioner and public education to encourage appropriate antibiotic use. 26 KAP were included in the review and 16 were meta-analysable. KAP respondents in Australia and the UK are consistently more likely to report beliefs and behaviours that are not aligned with appropriate antibiotic use, compared to participants in similar studies conducted in Sweden.

**Conclusions:**

Interactions between public knowledge, attitudes and their impacts on behaviours surrounding community use of antibiotics are complex and contingent. Despite a greater focus on raising public awareness in Australia and the UK, neither antibiotic consumption nor community knowledge and attitudes are changing significantly. Clearly public education campaigns can contribute to mitigating AMR. However, the relative success of policy approaches taken in Sweden suggests that practice level interventions may also be required to activate prescribers and the communities they serve to make substantive reductions in inappropriate antibiotic use.

## Introduction

Responding to the risk posed by antimicrobial resistance (AMR) became a key concern of governments, transnational organisations, and associated policy-makers at the turn of the century [1, 2]. The complexity of AMR and the extent to which antimicrobials have become embedded into the systems and structures that support our societies mean that AMR is a problem for which there is no simple or inexpensive solution [3–6]. Social and behavioural drivers of inappropriate antibiotic use have been identified as one of the key contributing factors to the emergence of AMR. In concrete terms, the need to address the drivers of AMR is frequently operationalised as requiring behaviour change from clinicians, patients, agricultural producers and consumers [7–9]. Therefore, enhancing the understanding of the broader public of the causes and consequences of AMR, and their role in minimising antibiotic misuse, is considered to be an important component of an effective and optimal public health response [10–14].

Since 2015, population-based knowledge, attitude and practices (KAP) surveys are part of the monitoring and evaluation framework proposed by the WHO Global Action Plan on Antimicrobial Resistance [12, 15]. Because of cross-national differences in antibiotic use, it is assumed that educational interventions need to be tailored to the needs of different social and cultural groups and audience in each country. As well as an individual’s knowledge about and attitudes towards antibiotics, their use has been found to vary because of other individual and cultural factors such as gender, education, age, tolerance of uncertainty, trust in health care providers and systems, and the level of corruption tolerated within governing institutions [16–21]. Local barriers and enablers to appropriate prescribing may partly explain some variations. For example, a busy practitioner may find it easier to prescribe a requested antibiotic than to attempt to educate the patient about why antibiotics are inappropriate for that condition [22]. KAP surveys endeavour to provide an understanding of some of the social drivers of inappropriate antibiotic use (either misuse or overuse) in the population. Aligned with the global action plan on AMR, it is proposed that surveillance of the levels of knowledge and awareness can contribute to the design of interventions, which can change the population’s behaviour on antibiotic use which could potentially lead to a reduction in AMR.

Previous systematic reviews and meta-analyses of public KAP around antibiotics are focused on studies published between 2000 and 2016 and have used either continents or global regions as the unit of analysis [23, 24]. Key findings include that lay-publics in Europe, Asia, the Americas and Oceania are generally aware of antibiotic resistance but have an incomplete understanding of and misperceptions about its causes and potential solutions [23, 24]. More recently, a systematic review of KAP survey design highlights that the association between the KAP of a population and the emergence of AMR in the community has not been clearly demonstrated [25]. Supporting qualitative research indicates that mistaken beliefs and a broader dissociation from the problem is being found in members of the public in Australia [10], the United Kingdom (UK) [26] and other jurisdictions [12, 23, 27]. At the same time, national differences in antibiotic prescription rates do not clearly correspond to the prevalence of bacterial infections [28, 29]. Instead, it is becoming increasingly clear that the rates of antibiotic consumption in the community is an outcome of national and local policies, accepted modes of treatment, the type of health-care system, and a number of cultural factors such as risk aversion [16–19]. Against a policy background within which variations in the rates of community use of antibiotics are substantial and remain difficult to explain [28, 30–32], evidence is mounting that efforts to educate the public are not producing the desired results [13, 33].

This review aims to compare the outcomes of surveys of the general population’s levels of knowledge and awareness of AMR against policy approaches taken to address antibiotic misuse in nations with similar primary care systems but differing rates of community antibiotic consumption. We systematically reviewed and compared peer-reviewed KAP surveys conducted within Australia with similar surveys conducted in the UK and Sweden. Recent data indicates that Australia continues to have one of the highest rates of hospital and community use of antibiotics in the OECD [34]. Despite some recent modest improvements, in 2020 Australian hospitals prescribed more than twice the defined daily doses (DDD) per 1000 patients than hospitals in Sweden and about 10% more than hospitals in the UK; the Australian community also consumed more than twice the defined daily doses per 1000 population than in Sweden and about 20% more than in the UK [34, 35]. Studies of antibiotic use in the community, in Australia and the UK, strongly suggest high rates of inappropriate prescribing in primary care, particularly unnecessary use for self-limiting illnesses [36–38]. Whereas, from an international perspective antibiotic use in the community in Sweden is low—most recently measured at 11.8 DDD per 1000 people per day in 2019 [35].

Our research explores how measures of public knowledge, attitudes and practices surrounding antibiotics and AMR in these three settings might correspond to different policy approaches and healthcare contexts. This study therefore seeks to generate new insights as to the role of lay publics in changing how antibiotics are used and the importance and limitations of public education and awareness raising campaigns in generating this change. By use of a systematic approach, we try to give more comprehensive and contextualised information about the characteristics and outcomes of public knowledge about AMR in three comparable high-income countries in order to evaluate the available evidence on the impact of different policy and institutional factors to further understandings of how to improve community use of antibiotics.

### Policy and practice background in the three study settings

Health care in Sweden, the UK and Australia is mainly publicly financed with primary care organised around family doctors or general practitioners (GPs) who typically work in group practices. In Australia, there is no registration requirement to access primary care, so anyone can go and see any GP available; whereas in the UK and Sweden individuals need to register with a GP who becomes their primary care provider [39]. In Australia and the UK, GPs are re-imbursed on a per consultation basis with longer and/or more complex clinical encounters being paid a higher fee; whereas, in Sweden GPs are usually salaried employees who are paid for their time, and not on the basis of the number or length of consultations [40, 41]. Because primary care in Australia operates in an open-access and fee-for-service environment, arguably GPs in this setting may place a high value on rapport with their patients than in the UK or Sweden—for clinical, as well as financial reasons [42]. Against these background conditions, community access to antibiotics is controlled in each of these settings by a requirement for registered primary healthcare providers to prescribe antibiotics to individual patients. Prescriptions for antibiotics in Australia, the UK and Sweden are filled by community pharmacists, and costs publicly subsidised or protected by patient payment caps, with a small contribution to the cost being made by the patient.

#### Policy approaches to community use of antibiotics and AMR in Australia

In Australia, efforts to attenuate antibiotic misuse in the community have focused on awareness raising and education among practitioners and publics. The Australian response to AMR in the 2015–2019 national action emphasize communication and consensus among implicated stakeholders such that actions to address AMR in Australia have mainly been delegated non-government actors and organisations [43]. The National Prescribing Service (NPS)-MedicineWise has been the key agency overseeing the Australian response to AMR in primary care [34]. NPS-MedicineWise have instantiated several AMR focused programs for clinicians including: using information products through mail-outs; voluntary participation for practitioners in face-to-face educational outreach and feedback from clinical auditing [44]; and, providing access to passively collected resistance surveillance data by collation of clinically submitted samples–noting that most antibiotic are prescribed empirically without testing [42]. Noting that unlike the UK and Sweden, mandatory clinical auditing and feedback, pay for performance measures and prescribing targets have not been put in place.

In Australia, campaigns targeting the public began in the early 2000s deploying marketing techniques, such as public posters, articles in printed news media and interviews in local and national radio to raise community awareness about AMR and encourage people not to seek antibiotics when experiencing a cold or flu [45]. NPS MedicineWise also conducted several consumer campaigns to change these community expectations, using simple advertising and innovative social media campaigns which have had some impacts to attenuate antibiotic use in the Australian community [44]. But, arguably, given the slow decline of otherwise sustained high rates of antibiotic use in the community, the impact public awareness raising and prescriber education has been modest [46].

#### Policy approaches to community use of antibiotics and AMR in the UK

Since the mid-2000s, the UK response to AMR situates the UK government as an active leader in developing solutions to the issue, particularly through establishing formal mechanisms that hold relevant national bodies and implicated stakeholders and professional groups to account [47]. Building on previous national plans, the UK strategy also assigns proposed actions to relevant government departments or agencies; thus providing clear mechanisms for departmental accountability [48]. For primary care clinicians the focus has been on improving antibiotic prescribing through: providing access to local prescribing and resistance data; and, the development and implementation of formal quality indicators of appropriate prescribing [22]. Since 2015, the UK has financially rewarded local clinical governance groups to reduce the amount of antibiotic prescribed in primary care in their jurisdictions which, it is claimed, has led to a decrease in community antibiotic use [49, 50].

At the same time, the UK had several campaigns to raise public awareness, including digital and social media, citizen science, Science-Arts and community theatre projects and more traditional advertising, but the impacts of most interventions have not been measured and are therefore unknown [51]. Public awareness interventions that have been evaluated have used mass media to promote rational antibiotic use with mixed results, with some interventions driving improvements in public knowledge [52], others showing no positive effects and one even increasing the likelihood of self-medication and non-rational use [9, 27]. After earlier improvements in prescribing rates and levels of AMR in the community from substantial efforts in the previous decades [53], the UK is beginning to have increasing rates of poor quality antibiotic use [31].

#### Policy approaches to community use of antibiotics and AMR in Sweden

Sweden has led the way internationally in reducing antibiotic consumption in the community [31]. In Sweden, the focus was primarily on changing the behaviour of prescribers, with the knowledge and behaviour of patients and publics a secondary concern [54, 55]. In the mid-1990s, Swedish governments and health authorities took a bottom up regulatory approach to the risks of AMR by establishing the The Swedish Strategic Programme Against Antibiotic Resistance (STRAMA). STRAMA has two levels–a network of independent locally-focused multidisciplinary groups that provide prescribers with feedback and implement guidelines; and a national executive working group block funded by the government to develop and oversee the implementation of national strategies [55].

Through the STRAMA program a suite of measures to improve clinician prescribing was introduced which included: point prevalence and point of prescription surveillance systems that allow regulators to benchmark and then target and locally adapt guidelines on antibiotic use; and, restricting reimbursements to both patients and providers, working directly with prescribing healthcare professionals [12, 56]. Since the early 2000s, these measure have been bolstered by pay-for-performance measures that reward appropriate stewardship among health practitioners [57]. These efforts to track and control antibiotic use were complemented by a public awareness campaign involving educational materials for members of the public focussing in the negative effects for the individual from unnecessary use of antibiotics. Further public engagement and education was sustained through making available to the public information local and national about trends in antibiotic use while also drawing attention to the economic consequences of antibiotic resistance [58]. Frequently held up as an exemplar of an effective policy response to the risks posed by AMR, data show that the gains made in Sweden are incremental, with community consumption of antibiotics steadily declining a few percentage points over many years [59].

In what follows we undertake a systematic literature review publics and meta-analysis of KAP survey among member of the public in each of these settings in order to evaluate the available evidence on the impact of different policy and institutional factors to further understandings of how to improve community use of antibiotics.

## Methods

The systematic literature review protocol was registered on the PROSPERO database (2020 CRD42020210455). This systematic review is reported following the Preferred Reporting Items for Systematic Reviews and Meta-Analyses (PRISMA) 2009 statement, which was current at the time the project was conceived [60].

### Inclusion criteria for systematic review

We included primary studies that conducted surveys using a structured questionnaire administered to a general population, which met the following criteria:

- Focused on public knowledge, attitudes, beliefs or behaviours in the general public
- Respondents included: patients, people in the community, general public.
- Conducted in Australia, UK, and Sweden and the Netherlands (however, we did not identify any studies meeting the inclusion criteria from the Netherlands).
- Used quantitative and mix-method studies, however the qualitative data reported in mixed methods studies was not included.
- Reported on one of the outcomes of interest under the Knowledge, Action, or Practice categories
- Studies did not need to meet any predetermined quality criteria to be eligible.

Studies were excluded if they were:

- Systematic reviews or scoping reviews
- Qualitative studies only
- Published in abstract-only form
- Included aged care resident participant populations
- Included respondents who have the authority to prescribe antibiotics, or students enrolled in professional programs
- Conducted outside of Australia, UK, Sweden, or Netherlands
- Studies which did not include primary research data (e.g., an editorial or a letter)
- Studies that only included clinicians, did not measure knowledge or beliefs about antibiotic resistance or were published in abstract-only form were excluded.

### Search and information sources

We searched PubMed, EMBASE, PsycINFO, Web of Science and CINAHL for primary studies of any design that measured people’s knowledge, attitudes and beliefs about antibiotic resistance in Australia, the UK, Netherlands and Sweden published between January 1 2011 until July 30 2021. A PubMed search strategy was developed and adapted for other databases (S1 Fig). No restrictions were applied with regards to the language of publication. The search was not limited to age or gender group. As described in the PROSPERO Protocol, we planned to search the first 10 pages of Google Scholar using Keywords but did not conduct this search because of COVID-19 related limitations to our capacity.

### Study selection

Four reviewers (OH, TH, JM and CD) independently screened titles and abstracts in pairs, followed by full texts of relevant articles. Reviewer disagreements were resolved by re-review followed by discussion, or by referring to a third author.

### Data extraction

Data extraction was performed independently by four authors (OH, SM, AM, and BN) in pairs. Disagreements on data or significance was resolved with a discussion until consensus was reached or by referring to a third author.

The extracted quantitative data was synthesised by grouping similar fixed responses into categories. Because questions were not identical in the different studies, the information suitable for inclusion was extracted from relevant fixed responses and the percentage of participants who responded affirmatively to the provided statement (yes or strongly agree/agree). After an initial appraisal, ten statements that grouped questions that were common for the different studies were considered as outcomes for the meta-analyses:

1. Practice: I consumed an antibiotic (however obtained, for any reason) in the **last 12 months**
2. Practice: I took/gave/administered antibiotics for a **viral** infection (cold, flu, etc.)
3. Practice: I saved/kept/retained **leftover** antibiotic/s
4. Knowledge: I know that antibiotics are useful/effective for treating **bacterial** infections
5. Knowledge: I know that antibiotics are useful/effective for treating **viral** infections (flus, colds, etc.)
6. Knowledge: I know that antibiotics have **adverse events**/harms/side effects/complications
7. Knowledge: I know that excessive/overuse of antibiotics **impacts their effectiveness** (decreases their effectiveness)
8. Attitude: I would save/keep/retain the **leftover antibiotic/s**
9. Attitude: I would expect/want to receive antibiotics for a **viral** infection (cold, flu, etc.)
10. Attitude: I would go to **see another doctor** when their (first) doctor did not prescribe/provide antibiotics

### Assessment of the risk of bias

**Fig 1:**
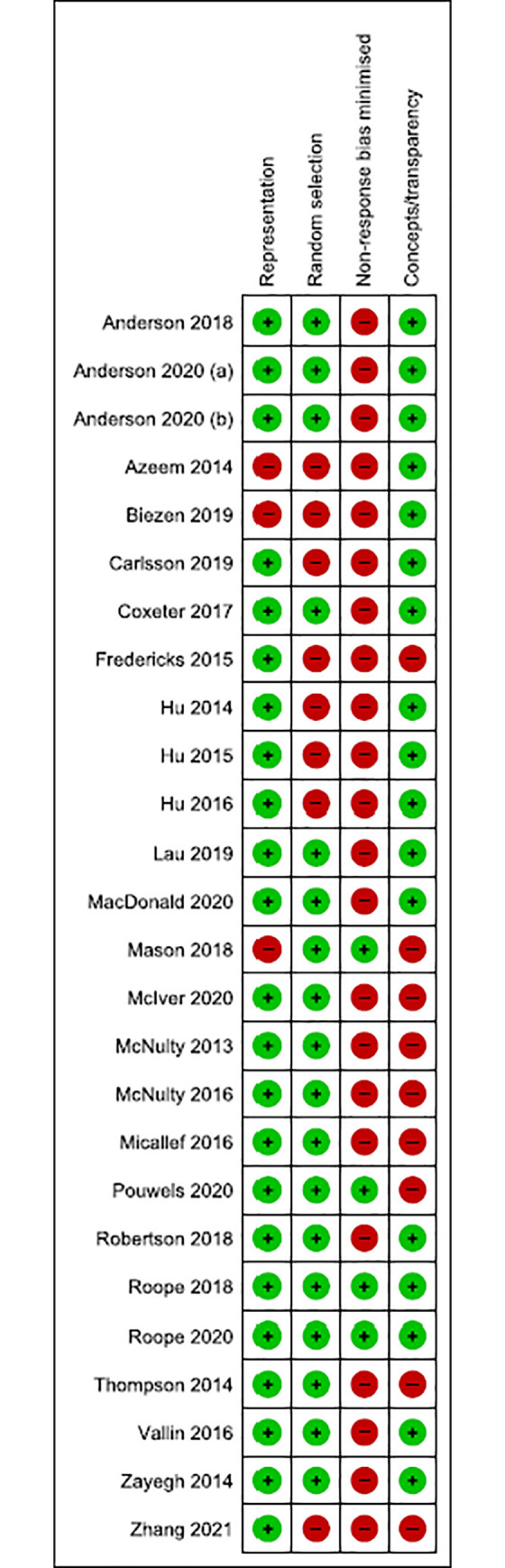
Risk of bias of the included studies.

The Risk of bias of the included studies was assessed by two authors independently in pairs (OH, SM, AM, BN). Disagreements were resolved by consensus or by referring to a third author. Risk of bias was assessed using a modified and shortened tool for assessing the risk of bias from Hoy and colleagues [61]. Each potential source of bias was rated as low or high (Fig 1). The following domains were assessed:

1. **Representation**: was the sampling frame a true or close representation of the target population?
2. **Random selection**: was some form of random selection used to select the sample (or was a census undertaken)?
3. **Non-response:** was the likelihood of non-response bias minimal?
4. **Concepts/transparency:** was the survey instrument or a list of interview questions provided in the study report?

### Measurement of effect and data synthesis

We calculated the percentage of respondents responding yes (or strongly and very strongly agree) to identical or very similar questions on a survey or questionnaire. We undertook meta-analyses only when meaningful (i.e. ≥2 studies or comparisons) data were available. Data were sub-grouped by country (Australia, Sweden, United Kingdom). Anticipating considerable heterogeneity, we used a random effects model. STATA (version 16.1) was used to perform the meta-analyses. Where survey data was collected from multiple waves (e.g. Pouwels et al., 2020, which was collected in 2015, 2016 and 2017), we used the data from a single, most recent survey wave for consistency (here, data from 2017).

## Results

### Results of the search

**Fig 2:**
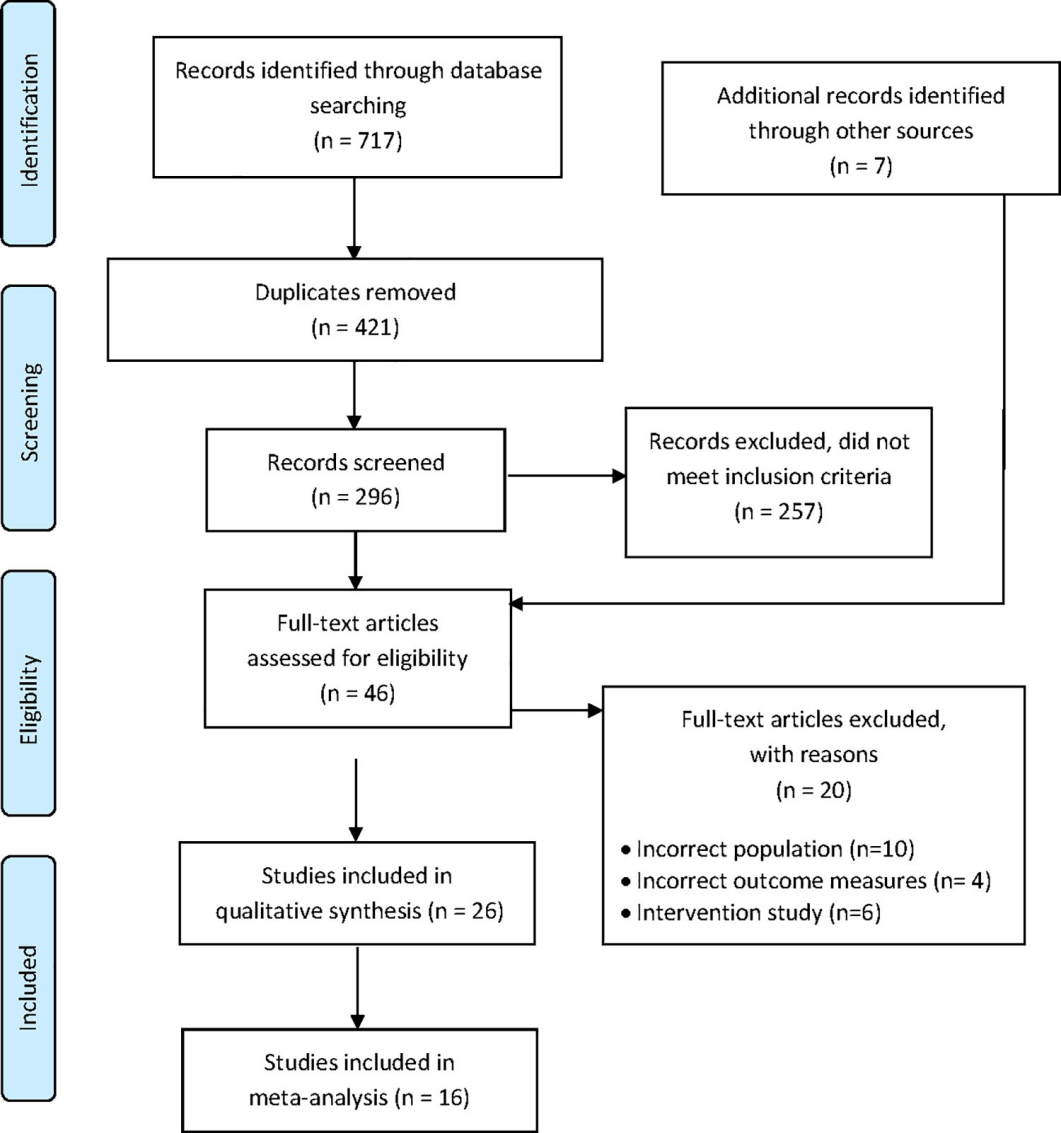
PRISMA flow diagram.

Database searches yielded 717 records. After removing 421 duplicates, 296 references were screened in title and abstract and 257 were excluded. Seven additional records were identified from searching the reference lists of included studies, which resulted in 46 references being screened in full-text. Twenty of these references were excluded with reasons (see Fig 2, and S1 Table). A total of 26 studies in the qualitative synthesis and 16 studies in the meta-analysis (see Fig 2).

### Characteristics of included studies

We included 26 studies, all of which were cross-sectional surveys (Table 1). 13 studies were conducted in Australia, 10 studies were conducted in the UK, and 3 studies were conducted in Sweden.

**Table 1:** Characteristics of included studies.

Author Year	Study location	Mode of study administration	Data collection period	Participants (respondents)	Number of participants	Age	Outcomes reported		
							Knowledge	Attitude or perception	Practice or behaviour
Anderson 2020 [62]	Australia	Online	2 weeks in July 2016	Parents or guardians of children <18yo	2157 parents (of 3971 children)	Range 18–71	✓	✓	✓
Azeem 2014 [63]	Australia	Self-administered	13–19 October 2013	Adults	224	Mean 42.4 (±12.4)	✓	✓	✓
Biezen 2019 [64]	Australia	Self-administered	June 2014 and July 2015	Adults	20 (GPs)	Range 31–70 (GPs)	✓	✓	
					50 (Parents and carers)	Range 21–50+			
Coxeter 2017 [65]	Australia	Face to face	May toJune 2014	Parents or guardians of children 1–12 years	401	Range 26–55	✓		✓
Fredericks 2015 [66]	Australia	Self-administered	July to October 2012	Pharmacy consumers	252	Range 18–75+	✓	✓	
Hu 2014 [67]	Australia	Online	July to October 2013	Australian Chinese migrants	426	Range 14–63		✓	✓
Hu 2015 [68]	Australia	Online	July to October 2013	Australian Chinese migrants	417	Range 14–63			✓
Hu 2016 [69]	Australia	Online	July to October 2013	Australian Chinese migrants	426	Range 14–63		✓	✓
Lau 2019 [70]	Australia	Self-administered	April to December 2015	Adults outpatients	68	Range 42–88			✓
McIver 2020 [71]	Australia	Face to face	March & August 2018	Adults	954	Range 18+		✓	
Thompson 2014 [72]	Australia	Face to face	N/R	Pharmacy consumers	57	N/R	✓	✓	
Zayegh 2014 [73]	Australia	Self-administered	February to May 2012	Pharmacy consumers	123	Range 18–80+	✓	✓	✓
Zhang 2021 [74]	Australia	Online	16 March—1 April 2020	General public	2217	Range 18+	✓		
Anderson 2018 [75]	UK	Face to face	2 June 2015–1 Nov 2015	Adults	1524	Range 32–63	✓	✓	✓
Anderson 2020 [76]	UK	Online	2 weeks in July 2016	Adults	2016	Range 15–67+	✓	✓	✓
MacDonald 2020 [77]	UK	Online	N/R	Adults	402	Range 35–82	✓	✓	
Mason 2018 [78]	UK	Self-administered	July 2014 and February 2015	Adults: affluent and deprived areas	139 (affluent) 220 (deprived)	N/R	✓		
McNulty 2013 [79]	UK	Face to face	January 2011	General public	1767	Range 15+			✓
McNulty 2016 [80]	UK	Face to face	January 2014	General public	1625	Range 15+	✓		✓
Micallef 2016 [81]	UK	Face to face	18 November 2015	General public	145	Range 15+	✓		
Pouwels 2020 [82]	UK	Online	1) May/June 2015	General public	10,064	1) Mean 44.2 (±15.7)	✓	✓	
			2) Oct/Nov 2016			3) Mean 46.6 (±16.9)			
			3) Mar 2017						
						3) Mean 46.5 (±16.8)			
Roope 2018 [83]	UK	Online	May-June 2015	Adults	2064	Mean 44 (±15.7)	✓	✓	✓
Roope 2020 [84]	UK	Online	Wave 1: Oct & Nov 2016; Wave 2: Mar 2017	Adults	4000 (Wave 1)	Wave 1: N/R	✓	✓	
					4000 (Wave 2)	Wave 2: Mean 47.2 (±16.7)			
Carlsson 2019 [85]	Sweden	Online	March 22—April 16 2017	Adults	1906	Range 18–75	✓	✓	✓
Robertson 2018 [54]	Sweden	Self-administered	N/R	General public	1293 (cross-sectional study) 3605 (‘Trust’ survey)	Range 16–85	✓	✓	
Vallin 2016 [18]	Sweden	Self-administered	2013	Adults	1426	Range 18–74	✓	✓	

### Risk of bias

Risk of bias was low for the representation and random selection domains, for most of the included studies. Nearly all studies were rated at high risk of for the non-response bias domain, and two-thirds were at low risk of bias for the concepts/transparency domain.

### Practice outcomes (action or behaviour)

#### 1). I consumed an antibiotic (however obtained, for any reason at all) in the last 12 months

**Fig 3:**
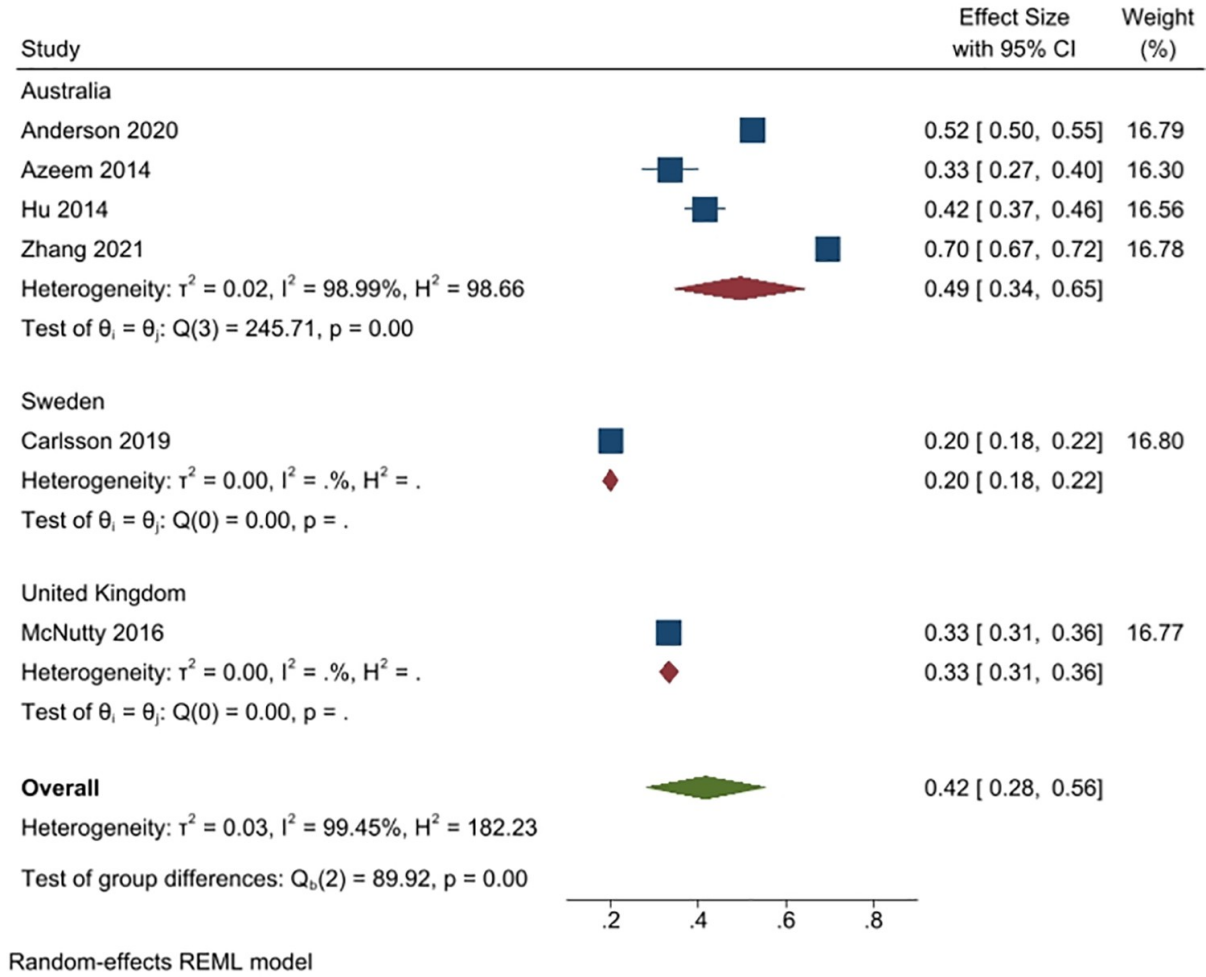
Percentage of respondents answering yes to the question of whether they consumed an antibiotic in the previous 12 months.

Six studies (8096 people) reported on what percent of respondents reported either consuming an antimicrobial themselves or administering it to someone in their care, for any reason, in the previous 12 months (4 studies from Australia, n = 5025; 1 from Sweden, n = 1906; and, 1 from the UK, n = 1625) Overall, 42% of people took antimicrobials in the previous 12 months (95% CI: 28% to 56%), although the heterogeneity of the pooled studies was very high (I^2^ = 99%). More Australian respondents (49%, 4 studies) reported consuming antibiotics than Swedish respondents (20%, 1 study) or UK respondents (33%, 1 study) (Fig 3).

#### 2). I took/gave/administered AB for a viral infection (e.g., cold, flu, etc.)

**Fig 4:**
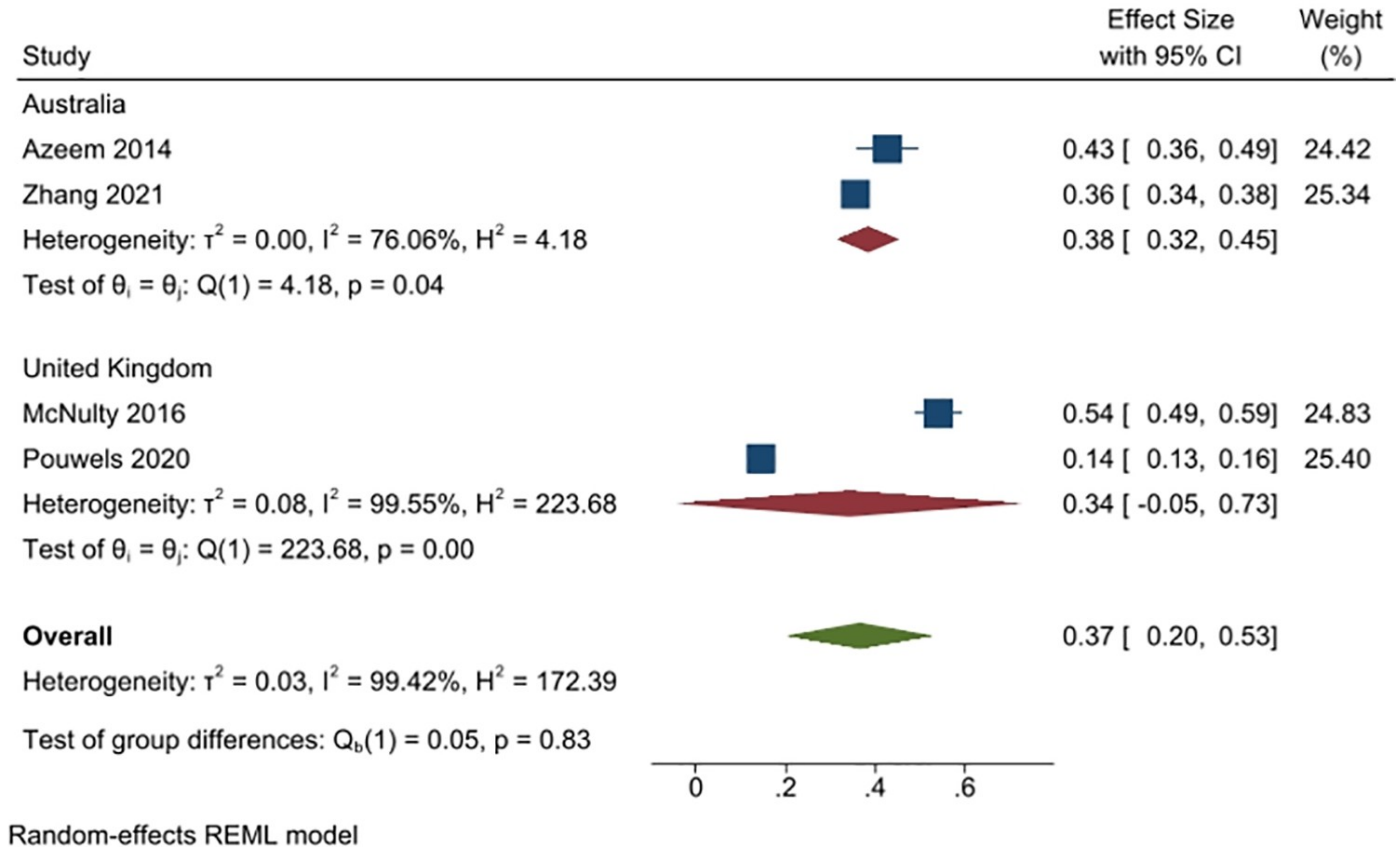
Percentage of respondents answering yes to the question of whether they took or administered an antibiotic for a viral infection.

Four studies (6804 people) reported on the percent of respondents who either consumed themselves or administered to someone in their care, an antibiotic for a viral infection (2 studies from Australia, n = 2441; and, 2 from the UK, n = 11689). Overall, 37% of respondents reported this (95% CI: 20% to 53%), with slightly more respondents in Australia reporting this (38%, 2 studies) than in the UK (34%, 2 studies). Heterogeneity was overall very high (I^2^ = 99%) (Fig 4).

#### 3). I saved/kept/retained leftover antibiotic/s

**Fig 5:**
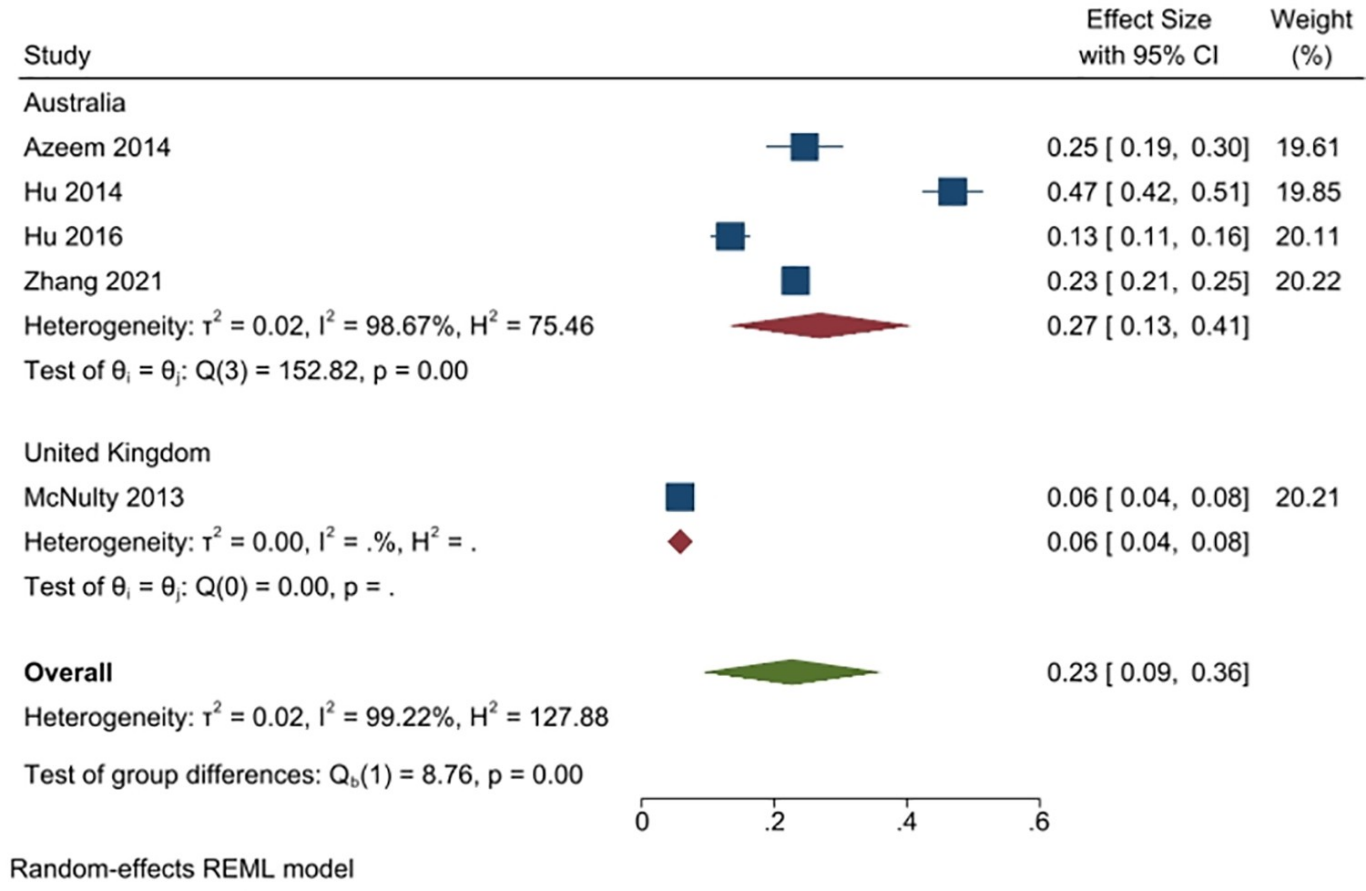
Percentage of respondents answering yes to the question of whether they retained leftover antibiotics.

Five studies (4039 people) reported on the percent of respondents who retained leftover antibiotics (4 studies from Australia, n = 3284; and 1 from the UK, n = 1767). Overall, 23% of respondents (95% CI: 9% to 36%) reported doing this, with the percentage higher in Australia (overall average 27%, 4 studies) than in the UK (6%, reported by 1 study). Heterogeneity was overall very high (I^2^ = 99%) (Fig 5).

### Knowledge outcomes

#### 1). I know that antibiotics are useful/effective for treating bacterial infections

**Fig 6:**
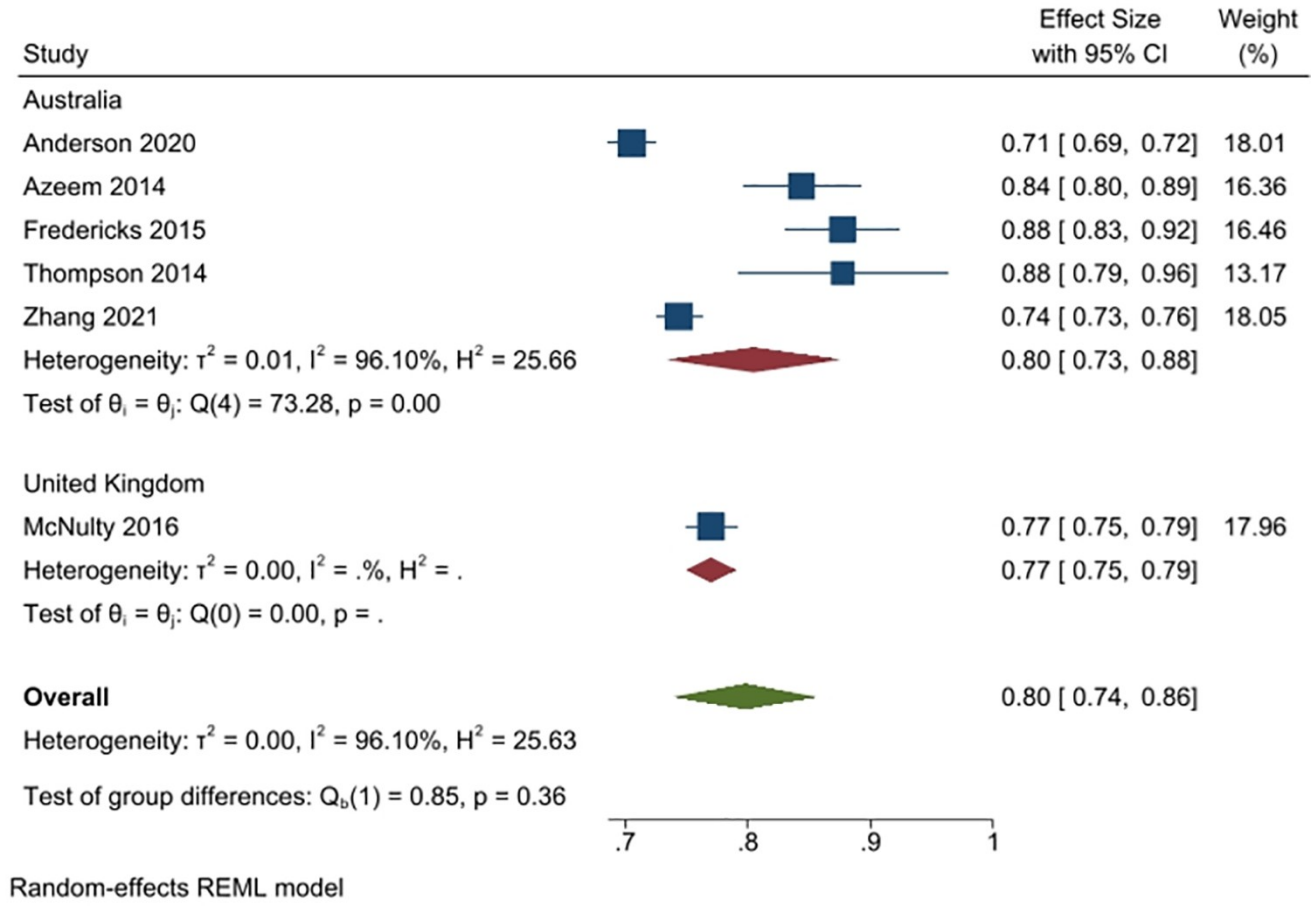
Percentage of respondents answering yes to the question whether antibiotics are useful or effective for treating bacterial infections.

In total, 6 studies (n = 6475 people) asked the respondents whether antibiotics are useful (or effective) for treating bacterial infections (5 studies from Australia, n = 4907; and 1 from the UK, n = 1625). Overall, 80% of respondents (95% CI: 74% to 86%) reported that antibiotics are useful for treating bacterial infections, with the percent higher in Australia (80%, 4 studies) than in the UK (77%, 1 study). Heterogeneity was very high (I^2^ = 96%) (Fig 6).

#### 2). I know that antibiotics are useful/effective for treating viral infections (e.g., flus, colds, etc.)

**Fig 7:**
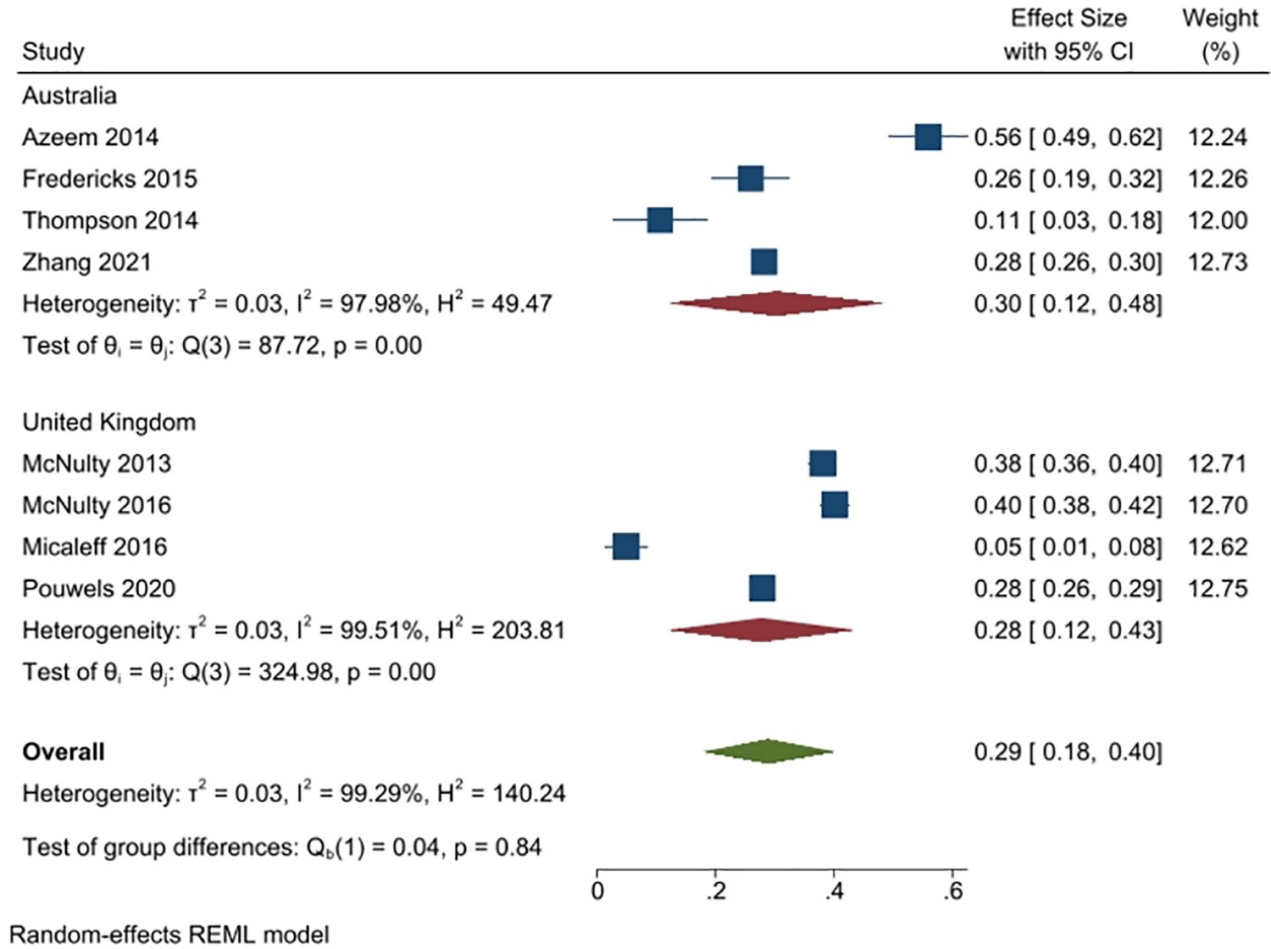
Percentage of respondents answering yes to the question whether antibiotics are useful or effective for treating viral infections.

Eight studies (n = 10,213 people), from Australia (4 studies, n = 2750) and the UK (4 studies, n = 13601) asked the respondents whether antibiotics are useful or effective for viral infections, and were able to be pooled. Overall, 29% respondents said antibiotics are useful for viral infections (95% CI: 18% to 40%). The responses were similar for the Australian respondents (30%, 95% CI: 12% to 48%) and the UK respondents (28%, 95% CI: 12% to 43%). Heterogeneity was very high (I^2^ = 99%) (Fig 7).

#### 3). I know that antibiotics have adverse events/harms/side effects/complications

**Fig 8:**
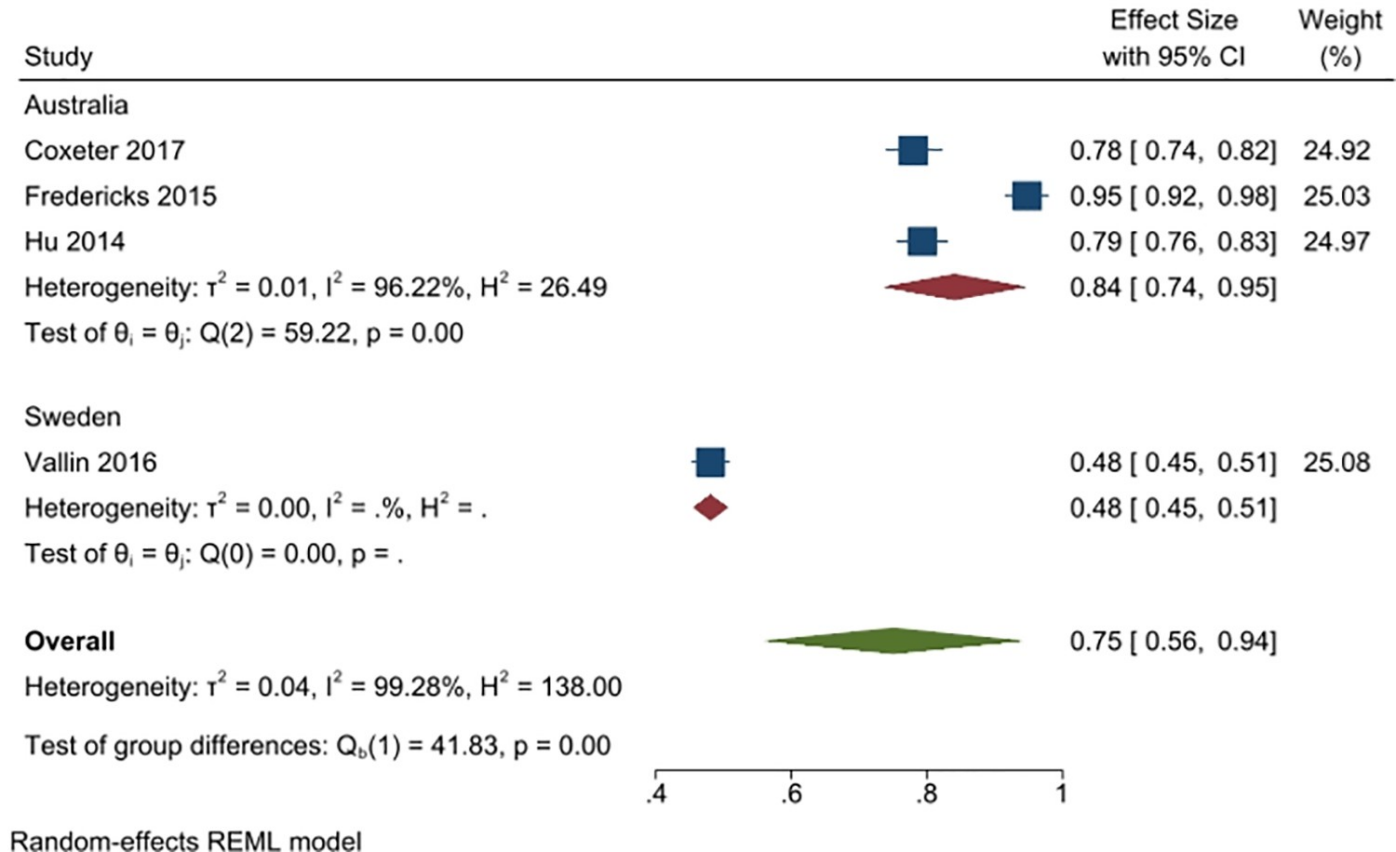
Percentage of respondents answering yes to the question whether antibiotics have adverse events or harms associated with their use.

Four studies (2471 people) asked respondents whether they know that antibiotics have adverse effects or harms/complications (3 studies from Australia, n = 1079; and 1 from Sweden, n = 1426). Overall, 75% responded yes (95% CI: 56% to 94%), with considerably more respondents in Australia (84%) than in Sweden (48%) responding in the affirmative. Heterogeneity was very high (I^2^ = 99%) (Fig 8).

#### 4). I know that excessive/overuse of antibiotics impacts their effectiveness (decreases their effectiveness)

**Fig 9:**
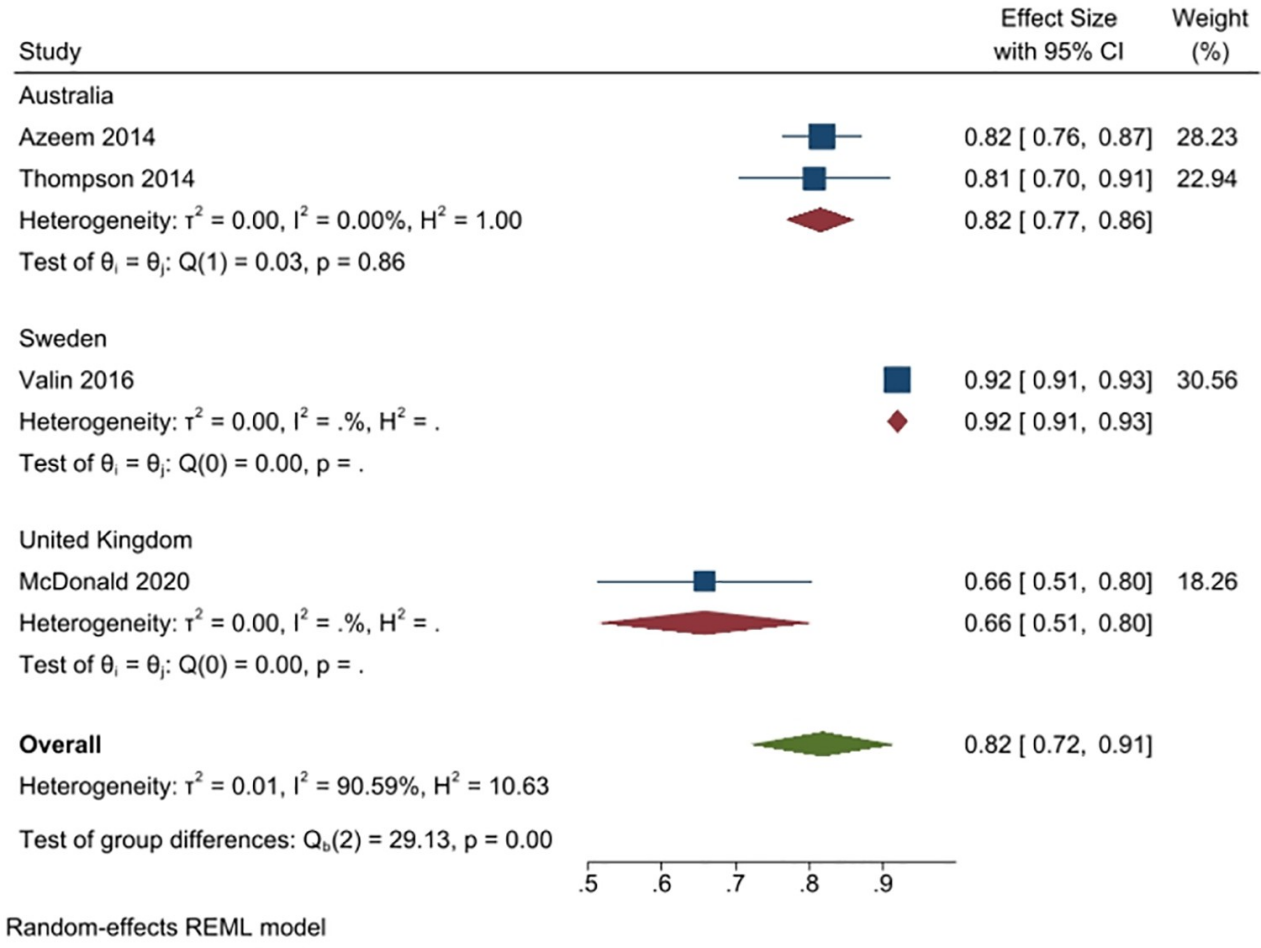
Percentage of respondents answering yes to the question whether antibiotic overuse impacts or decreases their effectiveness.

Four studies (1714 people) asked the respondents to answer a question on whether overuse of antibiotics affects their effectiveness (2 studies from Australia, n = 281; 1 from Sweden, n = 1426; and, 1 from the UK, n = 402). Overall, 82% of respondents responded affirmatively (95% CI: 72% to 91%), with the highest percentage responding affirmatively in Sweden (92%, 1 study), and the lowest in the UK (66%, 1 study); 82% of Australian respondents responded affirmatively. Heterogeneity was very high (I^2^ = 91%) (Fig 9).

### Attitude outcomes (belief, perception)

#### 1). I would save/keep/retain the leftover antibiotic/s

**Fig 10:**
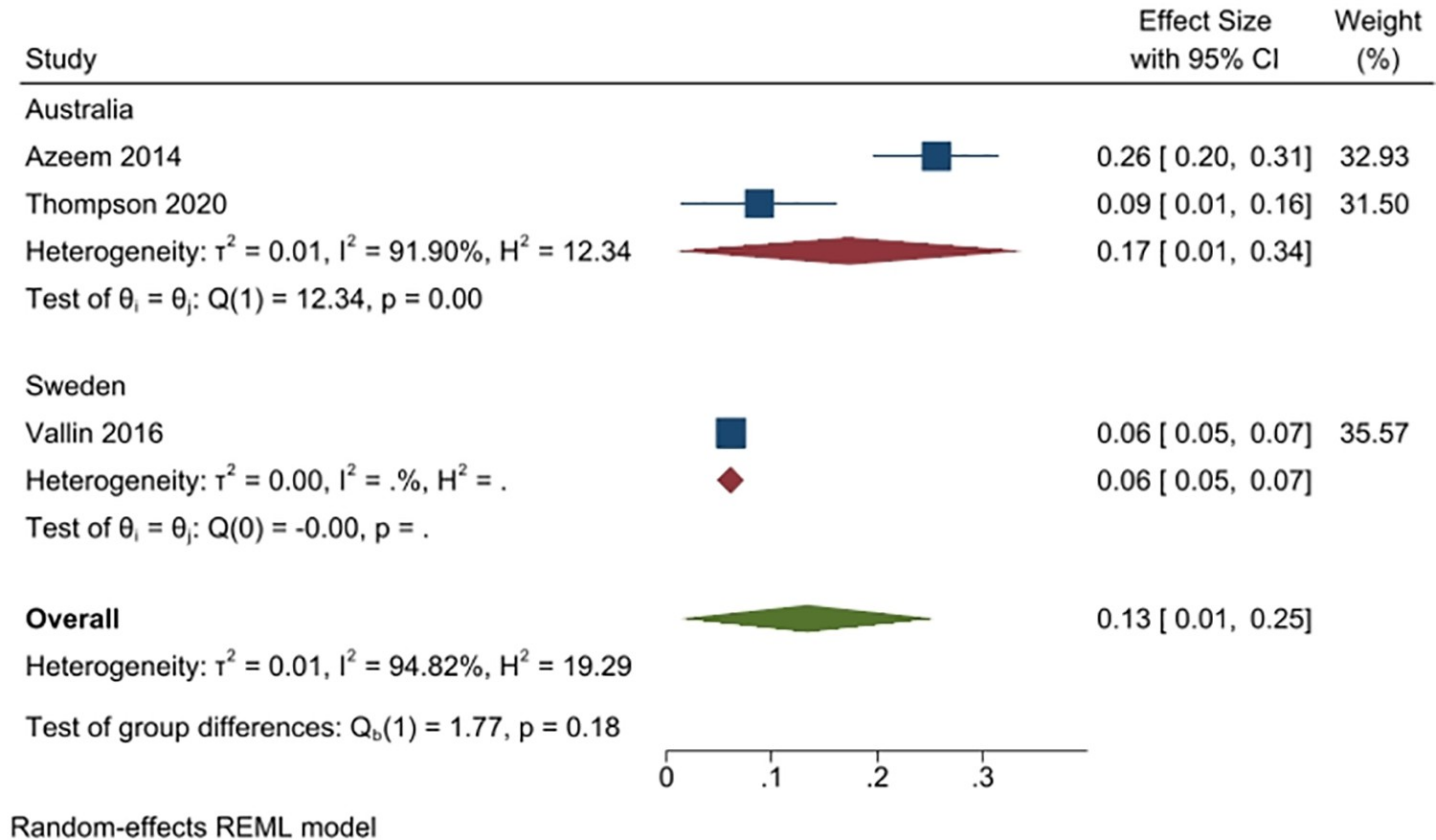
Percentage of respondents answering yes to the question whether they would retain leftover antibiotic.

Three studies (1680 people) asked respondents whether they would retain leftover antibiotics (2 studies from Australia, n = 281; and 1 from Sweden, n = 1426). Overall, 13% of respondents answered yes (95% CI: 1% to 25%), with fewer respondents in Sweden (6%) than in Australia (17%) answering yes. Heterogeneity was very high (I^2^ = 95%) (Fig 10).

#### 2). I would expect/want to receive antibiotics for a viral infection (cold, flu, etc.)

**Fig 11:**
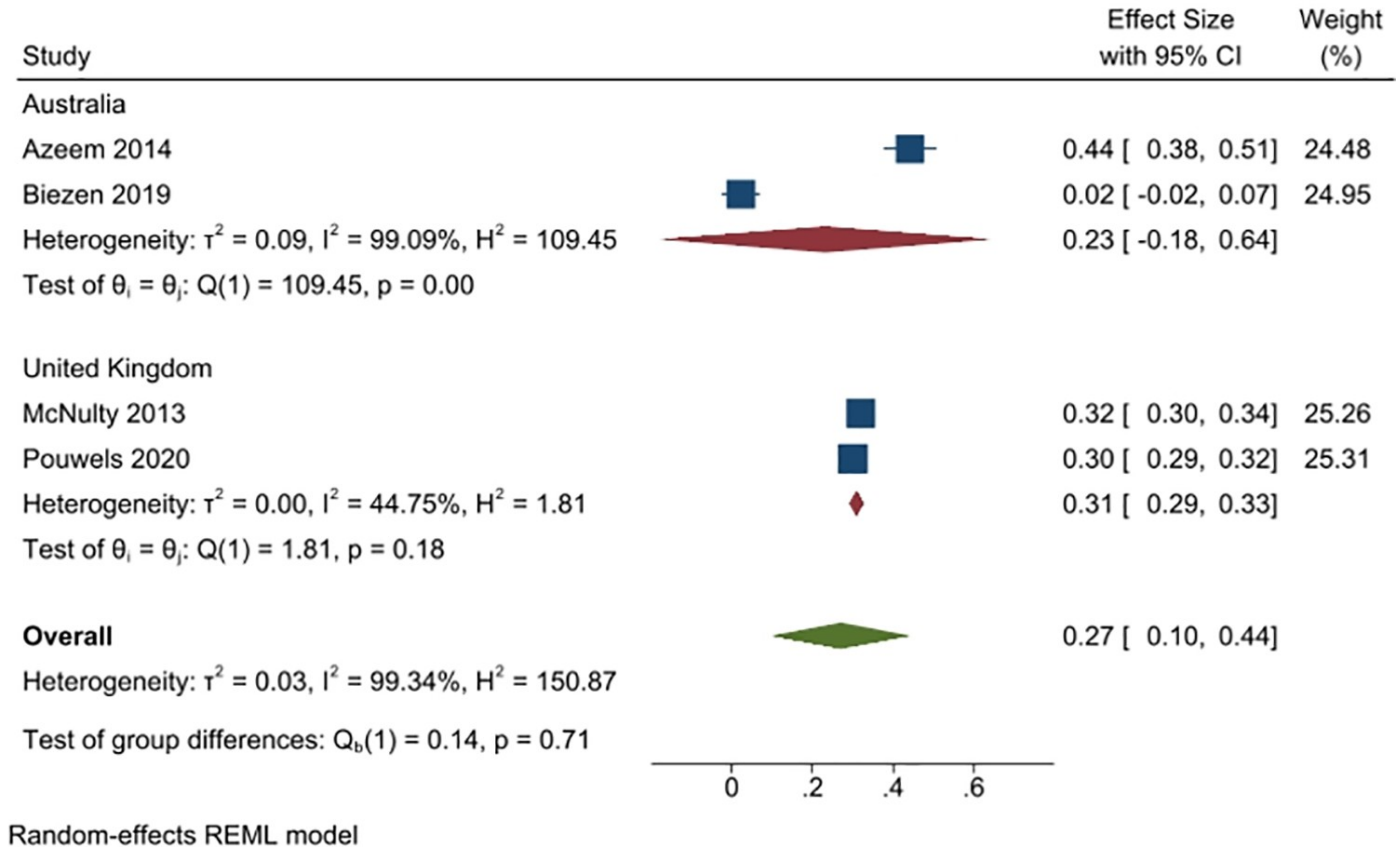
Percentage of respondents answering yes to the question whether they would expect or want to receive antibiotics for a viral infection.

Four studies (5271 people), asked respondents if they would want or expect to receive antibiotics for a viral infection (e.g. flu or cold) (2 studies from Australia, n = 274; and two from the UK, n = 11780). Overall, 27% of respondents answered yes (95% CI: 10% to 44%), with a greater proportion of UK respondents saying yes (31%, 2 studies) than Australian respondents (23%, 2 studies) (Fig 11).

#### 3). I would go to see another doctor when their (first) doctor did not prescribe/provide antibiotics

**Fig 12:**
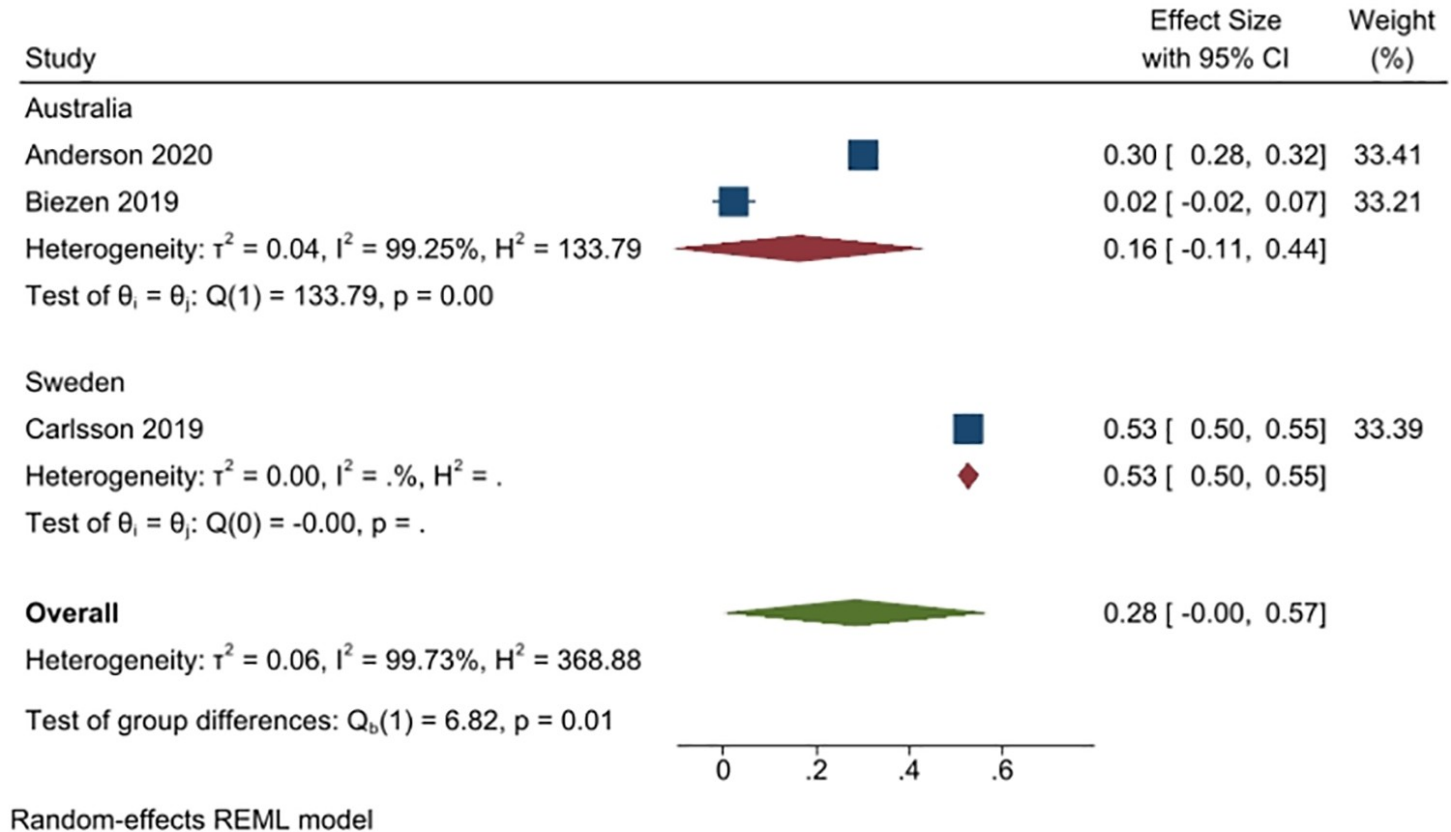
Percentage of respondents answering yes to the question whether they would see another doctor if their doctor did not prescribe an antibiotic.

Three studies (4410 people) asked respondents whether they would see another doctor, if the doctor they visited did not prescribe antibiotics (2 studies from Australia, n = 2207; and 1 from Sweden, n = 1906). Overall, 28% of respondents said yes (95% CI: 0% to 57%), with more respondents in Sweden (53%, 1 study), than in Australia (16%, 2 studies) responding in the affirmative. Heterogeneity was very high (I^2^ = 99%) (Fig 12).

## Discussion/Conclusions

Our systematic review and meta-analyses shows that against a policy background in Australia where the focus has been on educating practitioners and members of the public, lay-people who participate in KAP surveys report greater use of antibiotics than comparable studies in the UK and Sweden. The relative scale of these self-reported rates of consumption are consistent with other more formal measures of community use in each of these setting [34, 35]. Knowledge about the effectiveness of antibiotics for treating bacterial infections was high in both Australia and the UK at about 77–80% which is comparable to that found in the survey conducted by Andre and colleagues in Sweden in 2010 [86]. Results from previous systematic reviews and cross-sectional surveys consistently indicate that the capacity to engage with the biological aspects of AMR is strongly associated with educational attainment [23, 24, 75]. However, acknowledging the potential impacts of framing effects in how questions were asked in different surveys, when it comes to measures of attitudes and behaviours, participants in Australian and UK studies were consistently more likely to report beliefs and practices that are not aligned with appropriate antibiotic use when compared to the outcomes of similar studies conducted in Sweden.

Key outliers to this larger trend is that significantly more Australian survey participants reported keeping unused antibiotics than UK participants. The UK now has a self-reported antibiotic retention rate of 7% [79], that is comparable to a survey of the general public in 2010 in Sweden [86]. Similar levels of misunderstanding of the effectiveness of antibiotics against viral infections is reported in Australia and the UK–at around 30% which is comparable to that found in the survey conducted in Sweden at the beginning of the decade [86]. When it comes to public expectations for being prescribed antibiotics, similar attitudes were reported in Australia and the UK with a decrease in affirmative responses being seen in the former over the decade, and no real change in the later. Moreover, it is notable that the percentage of respondents reporting use of antibiotics to treat viral infections has declined markedly between the two surveys conducted in the UK [80, 82] in the period under review (40%), with only marginal changes over the same period found in the two surveys conducted in Australia [63, 74]. Participants in studies in Australia [65, 66] were much more aware than participants of study in Sweden [18] of the potential for antibiotics to cause side effects. Whereas Swedish [18] and Australian participants [63, 72] have a better knowledge of the use antibiotics being a key driver of AMR than their counterparts in the UK [77]. Perhaps reflecting the extent to which choices around primary care providers are restricted, survey participants in Sweden [85] were more likely than participants in Australia [62, 64] to prefer to see a second doctor if the first refused a request for antibiotics.

These differences in knowledge, attitudes and practices may reflect the varying impacts of different types of public awareness campaigns in these settings, but substantively, they are also somewhat at odds with accounts of the key messages and emphasis of each nation’s public communication strategy. In Australia, the focus has been on discouraging public expectations that antibiotics are needed to treat the common cold and influenza [13, 46]. Whereas the UK campaigns have provided the public with information about how to take antibiotics appropriately, alongside information about the consequences of inappropriate use of antibiotics [27, 87]. In Sweden, where public education has been given a relatively lower priority by health authorities, communications focused on the negative effects for the individuals and the broader costs to the health system of the unnecessary use of antibiotics [55, 56]. These differences also highlight how the standard justifications for and impacts of public awareness campaigns can create a causality loop where the underlying relationship between the knowledge, attitudes and practices of individuals and their populations are unclear. For example, the generally higher levels of knowledge and lower rates of antibiotic consumption in the community in Sweden suggests that public awareness raising and education should make a substantive difference in communities and settings where these measures are consistently lower. However the variations describe in this review highlight how interactions between knowledge, attitudes and their impacts on behavior and practice are complex and contingent [75, 78]. Health communications about AMR can spread inequitably through a population, such even as awareness is increased there is limited effect on attitudes and behaviours [88]. Despite the increasing focus on monitoring public knowledge and perceptions about antibiotic use and resistance in the UK and Australia, the high level of heterogeneity in our results suggest that without standardised survey instruments, directive and actionable information will remain elusive. Even as raising awareness among publics about AMR remains a central mechanism to address human behaviour, related research tends to focus on positive outcomes among intended audiences [89]. Moreover, our review did not identify and recent primary research on AMR-related KAP of the public in the Netherlands, even as the most recently reported DDD for this country is world-leading 9.1 per 1000 population [35].

Our findings point to the potential importance of regulatory environments for promoting appropriate antibiotic use in the community. In Australia, practice level policies have been oriented around voluntary engagement with educational messaging and materials. Over the same time period, health care providers in Sweden and the UK have had to adapt to responding to local fine detailed resistance and prescribing surveillance data supplemented by clinical auditing activities, targets and financial incentives and penalties. Internationally, a recent scoping review of antimicrobial stewardship in primary care highlights that in Australia and many other jurisdictions there is a lack of clear descriptions about who should be responsible for implementing and co-ordinating these activities [22]. In comparison to what has happened in the UK, the voluntary and consultative nature of the Australian government’s response to AMR risks has been characterized as a passive approach to AMR-related policymaking [90]. In Sweden’s STRAMA program and the UKs Clinical Consulting Groups, the pathways for the analysis and regular feedback to GPs are clear, as are the GPs’ responsibilities to act following feedback [49], and there is increasing evidence that strategies that reward appropriate prescriber behaviours have significant impacts on antibiotic use in the community [49, 57].

Field experiments in primary care settings in the US indicate that monetary incentive can enable providers to address the fixed costs (time or resources) of changing their practices [57, 91]. After having established a routine, another impetus is required to reverse it. This may explain why the effect often persists after the incentive is removed [92]. A recent qualitative study of the perspectives of Swedish primary healthcare providers notes that these reforms changed professional norms around prescribing, such that it became a collective enterprise that generated frustration with physicians who did not practice restrictive antibiotic use, or patients who insisted on antibiotics, or did not comply with given instructions about antibiotic use [93]. From the perspective of Swedish GPs, the health authorities and the media can play a large role in informing the public about the risks of antibiotic resistance, but it was the Swedish people who accepted and took on this responsibility, and ‘did their part’ in trying to reduce their use of antibiotics.

Finally, public health authorities and microbiologists have been advocating public education to promote more appropriate antibiotic since the early 1980s [1, 10]. However, recent social science scholarship draws attention to how the policy and institutional focus on KAP surveys as a measure and outcome of the communities response to AMR risks works to hide the structural drivers of antibiotic use [33, 88]. The reliance on KAP surveys in designing and evaluating public awareness campaigns make the problem of AMR one of individual and population knowledge deficits and compliance. This inevitably positions the regulatory environment and social dimension as secondary or passive influences on antibiotic consumption choices. The effect is that antibiotic consumption becomes decontextualized from factors that might limit the ability of individuals to choose differently, such as the system and structures through which health care and food are produced and can be accessed [3]. Critical analyses suggest that efforts to improve how members of the public in Australia and the UK use antibiotics are increasingly focused on nudging behaviorism, and creating social norms around good and bad behaviors, even as studies show that many people in these settings do not find the AMR problem to be personally relevant [33, 94]. Will [33] and Davis and colleagues [95] both suggest that the failure to engage broader publics in Australia and the UK could be a consequence of communication efforts that limit the scope of what lay-people can know and do, limiting opportunities for them to contribute to efforts to limit AMR. Lay publics have been brought into formal deliberative processes with other key stakeholders on how to instantiate cultural change to manage the drivers and impacts of AMR [96, 97], but these types of events are rare and have yet to be more broadly incorporated into related policy-making and resource allocations. At a minimum, broader bottom-up change could be initiated by messaging that elicit the public’s motivation to make individual and collective efforts to address AMR, now and in the future [95, 98].

### Strengths and limitations

Our study has several limitations. There was significant heterogeneity in outcomes of the surveys. There was also a limited number of studies reporting the outcomes in each of the 3 countries such that some of the meta-analyses only have 1 study for a specific outcome from a jurisdiction, and some outcomes have data from 2 but not all 3 countries. This is a limitation to the study as well as being a surprising finding, as both the UK and Sweden have been highly proactive in promoting antimicrobial stewardship for some time. The studies meeting the inclusion criteria were conducted with a broad range of participants (e.g., adults, parents, pharmacy consumers, migrants) with some variations in the research instruments used to collect data (e.g., responses to general surveys and vignettes), which may limit the generalisability of the findings to the general population. We believe that there would be considerable value in conducting a single multi-country survey across these settings, to mitigate some of these issues. We were also unable to identify any includable studies from The Netherlands, although we had intended to include studies from that country. There are also recognised limitations with AMR KAP instruments including that cross-sectional design is poorly oriented towards describing causal relationships [99], and that survey samples reliant on self-selection are likely to be biased towards good stewardship [76]. Gaining a better understanding of the importance and impact of public KAP about AMR relative to policy approaches and structural and system factors would require a study asking the same questions at the same time from each of those 3 countries.

The strengths of our study include that some survey outcomes are derived from pools of thousands of people, and there is a high level of consistency with other external measures of community knowledge, practices and behaviours such as antibiotic consumption [34, 35]. Previous systematic reviews and meta-analyses found a lack of knowledge about antibiotics among members of the public including indications for use (33%), their lack of efficacy against viruses (53.9%) and the role of antibiotics in driving AMR (26.9%) highlighting big differences between studies in different regions and jurisdictions [23, 24]. What our study shows is that such population level measures can be useful. But their interpretation needs to be grounded in context to develop approaches to public engagement with AMR that engender a strong civil society movement and political commitment. Future researchers should ensure that their study designs account for a reflect the conditions that structure what their participants can know and what actions their participants can realistically take that will contribute to broader efforts to reduce antibiotic consumption.

## Supporting information

S1 Checklist PRISMA 2009 checklist. (DOC)

S1 Fig Search terms/database search strings. (TIF)

S1 Table Table of excluded studies. (DOCX)

## List of abbreviations

AMR: Antimicrobial resistance
DDD: Defined daily dose
GP: General practitioner / family doctor
KAP: Knowledge, attitudes and practices
OECD: The Organisation for Economic Co-operation and Development
NPS: The National Prescribing Service
UK: United Kingdom
WHO: World Health Organization
