# Supplementary figures and images for: Comparing public attitudes, knowledge, beliefs and behaviours towards antibiotics and antimicrobial resistance in Australia, United Kingdom, and Sweden (2010-2021): A systematic review, meta-analysis, and comparative policy analysis
Source: PLoS One. 2022 Jan 14;17(1):e0261917. doi: 10.1371/journal.pone.0261917 (PMC8759643; doi:10.1371/journal.pone.0261917)

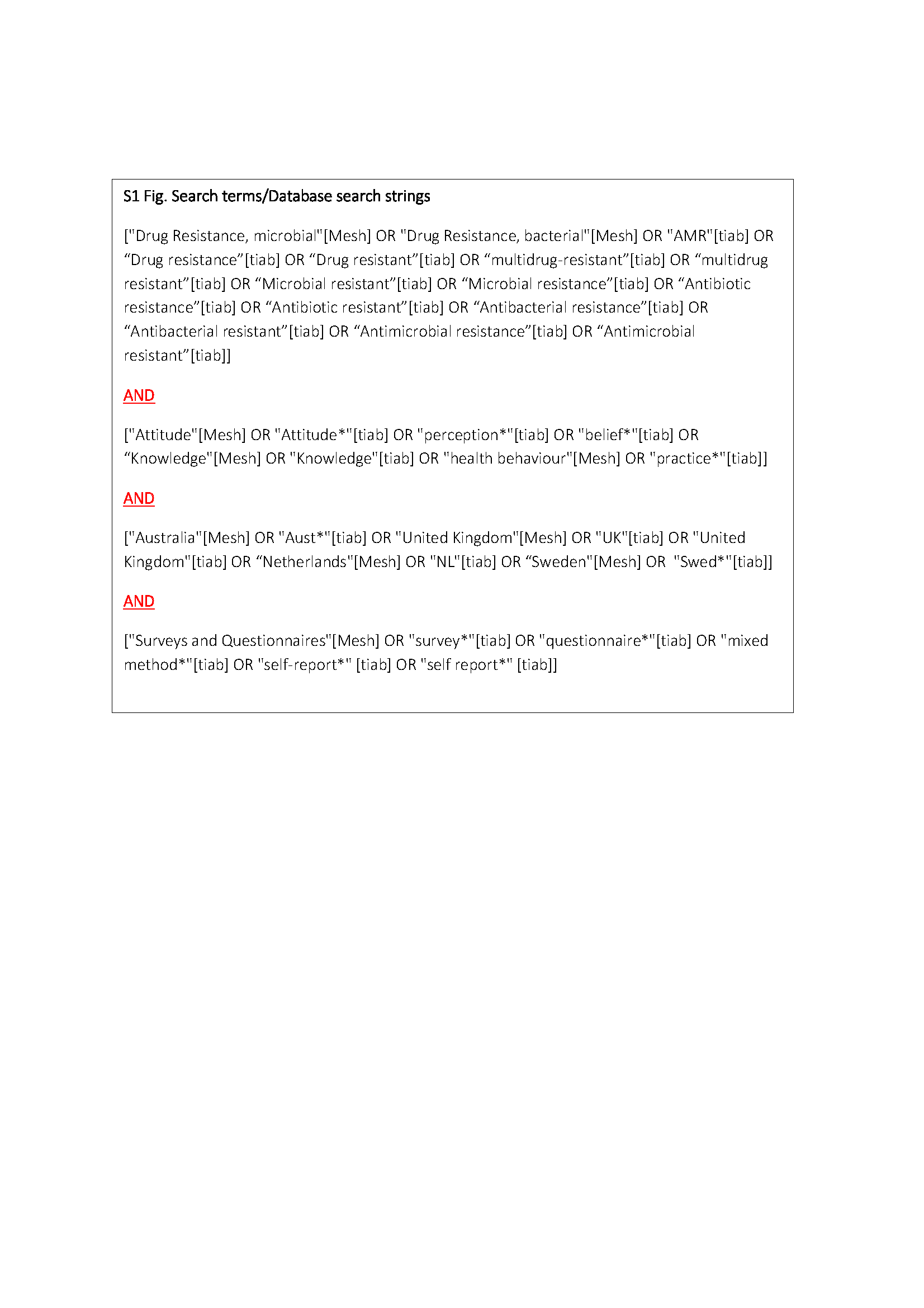

Supplement: S1 Fig — (TIF) [file pone.0261917.s002.tif]
