# Supplementary material for: Comparing public attitudes, knowledge, beliefs and behaviours towards antibiotics and antimicrobial resistance in Australia, United Kingdom, and Sweden (2010-2021): A systematic review, meta-analysis, and comparative policy analysis
Source: PLoS One. 2022 Jan 14;17(1):e0261917. doi: 10.1371/journal.pone.0261917 (PMC8759643; doi:10.1371/journal.pone.0261917)
Supplement: S1 Table — (DOCX) [file pone.0261917.s003.docx]

# S1 Table – Table of Excluded Studies

| **Reference** | **Reason for exclusion** |
| --- | --- |
| Ackah J, Neal L, Marshall N, Panahi P, Lloyd C, Rogers L. Antimicrobial prophylaxis in adult cardiac surgery in the United Kingdom and Republic of Ireland. Journal of infection prevention. 2021. | Population |
| af Sandeberg M, Johansson E, Wettergren L, Bjork O, Hertting O, Nilsson A. Antibiotic use during infectious episodes in the first 6 months of anticancer treatment A Swedish cohort study of children aged 7-16 years. Pediatric Blood & Cancer. 2017. | Population |
| Altorf-van der Kuil W, Schoffelen AF, de Greeff SC, Thijsen SF, Alblas HJ, Notermans DW, et al. National laboratory-based surveillance system for antimicrobial resistance: a successful tool to support the control of antimicrobial resistance in the Netherlands. Euro Surveill. 2017. | Intervention |
| Anderson A. Online health information and public knowledge, attitudes, and behaviours regarding antibiotics in the UK: Multiple regression analysis of wellcome monitor and eurobarometer data. PLoS ONE. 2018. | Outcome measures |
| Anderson A. Analysing incompliant attitudes towards antibiotic prescription completion in the UK. Journal of Antimicrobial Chemotherapy. 2020. | Outcome measures |
| Bausch K, Roth J, Seifert H, Widmer A. Overuse of antimicrobial prophylaxis in low-risk patients undergoing transurethral resection of the prostate. Swiss Medical Weekly. 2018. | Population |
| Bhattacharya A, Hopkins S, Sallis A, Budd E, Ashiru-Oredope D. A process evaluation of the UK-wide Antibiotic Guardian campaign: developing engagement on antimicrobial resistance. Journal of Public Health. 2017201. | Outcome measures |
| Chaintarli K, Ingle S, Bhattacharya A, Ashiru-Oredope D, Oliver I, Gobin M. Impact of a United Kingdom-wide campaign to tackle antimicrobial resistance on self-reported knowledge and behaviour change. BMC Public Health. 2016. | Intervention |
| Chan A, Horne R, Lycett H, Raebel E, Guitart J, Wildman E, et al. Changing Patient and Public Beliefs About Antimicrobials and Antimicrobial Resistance (AMR) Using a Brief Digital Intervention. Front Pharmacol. 2021. | Intervention |
| Coenen S, Francis N, Kelly M, Hood K, Nuttall J, Little P, et al. Are patient views about antibiotics related to clinician perceptions, management and outcome? A multi-country study in outpatients with acute cough. PloS one. 2013;8(10):e76691. | Intervention |
| Cotta M, Robertson M, Tacey M, Marshall C, Thursky K, Liew D, et al. Attitudes towards antimicrobial stewardship: results from a large private hospital in Australia. Healthcare Infection. 2014. | Population |
| Cuningham W, Anderson L, Bowen AC, Buising K, Connors C, Daveson K, et al. Antimicrobial stewardship in remote primary healthcare across Northern Australia. PeerJ. 2020. | Population |
| Ervin K, Tse KC, Reid C, Smith E. Exploring barriers to and enablers of antimicrobial stewardship in rural health services. Infection Disease & Health. 2021. | Population |
| Hall J, Jones L, Robertson G, Hiley R, Nathwani D, Perry MR. 'The Mould that Changed the World': Quantitative and qualitative evaluation of children's knowledge and motivation for behavioural change following participation in an antimicrobial resistance musical. PLoS One. 2020. | Intervention |
| Hobbs M, Grant C, Ritchie S, Chelimo C, Morton S, Berry S, et al. Antibiotic consumption by New Zealand children: exposure is near universal by the age of 5 years. Journal of Antimicrobial Chemotherapy. 2017. | Population |
| Hoffmann K, Ristl R, Heschl L, Stelzer D, Maier M. Antibiotics and their effects: what do patients know and what is their source of information? European Journal of Public Health. 2014. | Population |
| Islam J, Ashiru-Oredope D, Budd E, Howard P, Walker A, Hopkins S, et al. A national quality incentive scheme to reduce antibiotic overuse in hospitals: Evaluation of perceptions and impact. Journal of Antimicrobial Chemotherapy. 2018. | Population |
| Keizer J, Braakman-Jansen L, Kampmeier S, Koeck R, Al Naiemi N, Te Riet-Warning R, et al. Cross-border comparison of antimicrobial resistance (AMR) and AMR prevention measures: the healthcare workers' perspective. Antimicrobial Resistance and Infection Control. 2019. | Population |
| Lecky D, Hawking M, Verlander N, McNulty C. Using interactive family science shows to improve public knowledge on antibiotic resistance: does it work? PLoS One. 2014;9(8):e104556. | Intervention |
| Van Hecke O, Lee J, Butler C, Moore M, Tonkin-Crine S. Using evidence-based infographics to increase parents' understanding about antibiotic use and antibiotic resistance: A proof-of-concept study. JAC-Antimicrobial Resistance. 2020. | Outcome measures |
